# Transcriptional Bursting in Pluripotent Stem Cells

**DOI:** 10.3390/biology15120951

**Published:** 2026-06-18

**Authors:** Ruihe Lin, Yanhan Liu, Qiang Wu

**Affiliations:** 1 The State Key Laboratory of Mechanism and Quality of Chinese Medicine, Faculty of Chinese Medicine, Macau University of Science and Technology, Macau 999078, China; 3240006431@student.must.edu.mo; 2 The State Key Laboratory of Mechanism and Quality of Chinese Medicine, Faculty of Medicine, Macau University of Science and Technology, Macau 999078, China; xliuyouxiang@163.com; 3 The State Key Laboratory of Mechanism and Quality of Chinese Medicine, Macau University of Science and Technology, Macau 999078, China

**Keywords:** transcriptional bursting, pluripotent stem cells, bursting kinetics, transcriptional condensates, super-enhancers

## Abstract

Transcriptional bursting, the stochastic production of mRNA in episodic pulses, is a fundamental source of cell-to-cell heterogeneity. In pluripotent stem cells (PSCs), these bursting dynamics at core pluripotency loci are not just noise but critical determinants of identity maintenance and lineage commitment. This review synthesizes current quantitative frameworks for dissecting bursting kinetics and elaborates on the multilayered regulatory hierarchy that governs them, ranging from promoter-intrinsic features and 3D genome architecture to the formation of transcriptional condensates via liquid–liquid phase separation (LLPS). By integrating findings from genomic profiling and live-cell imaging, we highlight how the integrated action between super-enhancers and epigenetic states shapes the unique bursting dynamics in PSCs. Furthermore, we explore the functional consequences of these kinetics in pluripotency surveillance and cell fate decisions. Collectively, this review establishes a unified regulatory framework, providing novel insights for understanding stem cell heterogeneity and offering key insights for regenerative medicine.

## Simple Summary

Stem cells possess the unique ability to transform into various cell types, a process governed by how genes are activated. Instead of a steady flow, genes are often turned on and off in short, irregular pulses known as bursts. These bursts create natural differences between individual cells, helping stem cells remain flexible and ready to develop into specific tissues. However, the exact mechanisms controlling these pulses remain partially understood. This review explains how gene bursts are regulated by the physical arrangement of genetic material, chemical signals, and the cell’s energy use. We summarize how these pulses maintain the versatile state of stem cells and prepare them to become specialized tissues. By uncovering these hidden patterns of gene activity, this work provides a clearer map for using stem cells in medical research. Ultimately, understanding these processes will help scientists better grow and use stem cells to model diseases and develop new treatments for injuries and disorders in regenerative medicine.

## 1. Introduction

Transcription is the first step of gene expression, during which RNA polymerase synthesizes RNA from a DNA template. In eukaryotes, transcription initiation requires assembly of the pre-initiation complex at the promoter, recruitment and activation of RNA polymerase II, followed by promoter escape, elongation along the gene body, and eventual termination and RNA release [1,2]. Classical views treated this process as relatively continuous, producing RNA at a steady rate. However, single-cell and single-locus measurements have revealed that transcription is inherently stochastic and highly dynamic [3,4,5]. Many genes switch between active and inactive states, generating mRNA in short, intense episodes termed transcriptional bursts, separated by longer silent intervals [3,6]. These bursts, characterized by burst frequency and burst size, are now recognized as a pervasive mode of gene regulation from bacteria to mammals, and a major source of cell-to-cell heterogeneity in RNA and protein levels [6,7].

Pluripotent stem cells (PSCs) possess unique transcriptional properties that maintain self-renewal and developmental potency. Core pluripotency genes such as OCT4, SOX2, and NANOG are central to the maintenance of the pluripotent state in embryonic stem cells and induced pluripotent stem cells (iPSCs) [8,9]. Recent advances have revealed that these core genes display prominent transcriptional bursting, representing a classic paradigm for understanding gene expression heterogeneity in stem cells [10,11].

Transcriptional bursting at pluripotency loci is modulated by chromatin regulators [10], enhancer–promoter communication [12] and signaling pathways such as Akt/MAPK [13], which together tune burst size, burst frequency and promoter accessibility. Changes in bursting kinetics of core pluripotency genes, for example, reduced Sox2 burst size and frequency, are sufficient to trigger pluripotency loss [11], whereas appropriate control of variability contributes to a flexible yet robust pluripotent state [14]. Thus, transcriptional bursting is increasingly recognized not as mere noise, but as a critical regulatory mechanism that actively sustains pluripotency by shaping the molecular identity, functional heterogeneity, and developmental fate plasticity of PSCs [7,11].

Despite these advances, a comprehensive, integrated review of transcriptional bursting in PSCs, spanning from molecular mechanisms to functional outcomes, remains lacking. In this review, we systematically dissect the molecular mechanisms, upstream regulators, and functional outcomes of transcriptional bursting in PSCs. Moving beyond mere kinetic characterization, we integrate cutting-edge advances in phase separation, 3D genome architecture, and super-enhancer biology to elucidate how bursting shapes pluripotency maintenance, cellular heterogeneity, and differentiation readiness. By contextualizing PSC-specific bursting signatures within a unified regulatory framework, this work provides a foundational reference for future studies exploring transcriptional stochasticity in stem cell fate and regenerative medicine.

In this review, we distinguish three levels of evidence to avoid conflating speculative ideas with established mechanisms. “Established” findings refer to results supported by direct experimental manipulation in PSCs, such as acute perturbation of burst parameters combined with live-cell imaging or single-cell lineage tracing. “Correlative” evidence is inferred from single-cell transcriptomics, fixed-cell imaging, or genome-wide association studies without causal intervention. “Speculative” or “hypothetical” statements encompass theoretical models, extrapolations from non-PSC systems (e.g., yeast or bacteria), and untested proposals (for instance, putative roles of metabolic inputs or specific chromatin features). Throughout the text, we use corresponding language—such as “demonstrates” or “is sufficient to” for established findings; “suggests”, “correlates with”, or “is associated with” for correlative evidence; and “is hypothesized”, “a model proposes”, or “remains speculative” for hypothetical claims. Where direct evidence is missing, we explicitly flag this to highlight priorities for future investigation.

## 2. Fundamentals of Transcriptional Bursting in PSCs

### 2.1. Core Kinetic Parameters

#### 2.1.1. Transcriptional Noise

Gene expression in PSCs is highly variable and can be broken down into intrinsic and extrinsic noise components. Intrinsic noise stems from stochastic fluctuations in local promoter and allele-specific transcription, leading to burst-like expression heterogeneity [13,15]. Extrinsic noise, on the other hand, results from cell-to-cell variation in global factors like cell cycle stage, metabolic state and the abundance or activity of transcriptional machinery [16,17]. Identifying these components is crucial for understanding PSC heterogeneity, as intrinsic noise provides insights into promoter bursting dynamics, while extrinsic noise indicates broader cell-to-cell variation. The stochastic switching that drives intrinsic noise can be quantitatively captured by the two-state model, which defines several key burst parameters as described below.

#### 2.1.2. Burst Parameters

Under the classical two-state (telegraph) model of transcription, transcriptional bursting refers to the intermittent switching of genes between transcriptionally inactive (OFF) and active (ON) states. This model is commonly used to quantitatively describe and infer transcriptional bursting kinetic parameters due to its simplicity and excellent statistical fit to single-cell expression heterogeneity [7].

The model, depicted as a Markov process with constant rates, assumes that the gene transitions from OFF to ON at a constant rate kon (units: h^−1^) and from ON to OFF at rate koff (h^−1^). While in the ON state, RNA polymerase initiates transcription at a consistent rate γ (mRNA min^−1^ or h^−1^); in the OFF state, transcription initiation does not occur [18,19]. The following kinetic parameters define bursting dynamics:

- Burst duration: It refers to the average amount of time that a gene remains in the ON state during a single occurrence. Because the ON state terminates at a constant rate koff, the duration of the ON state follows an exponential distribution, with a mean value of:
  Ton=1/koff.
- Interburst interval and Burst frequency: The average interburst interval between consecutive bursts is:
  Tinter=1/kon+1/koff,
  taking into account the durations of both the OFF and ON states [20]. In most eukaryotic genes, the OFF duration is significantly longer than the ON duration (1/kon ≫ 1/koff) due to the substantial time required for chromatin remodeling and gene reactivation. In this scenario, the interburst interval simplifies to 1/kon, making the burst frequency, defined as the average number of transcriptional bursts per unit time, approximately equal to kon [19].
- Burst size: The average number of mRNA molecules produced during a single ON event. With a constant transcription initiation rate γ in the ON state and a mean ON duration of 1/koff [19,20], the burst size is:
  Burst size=γ/koff.

### 2.2. Key Methods to Quantify Bursting in PSCs

#### 2.2.1. smFISH and Single-Molecule Imaging

Single-molecule fluorescence in situ hybridization (smFISH) and single-molecule imaging quantify bursting in PSCs by counting individual mRNAs per cell and at active transcription sites. In smFISH, cells are fixed and permeabilized, then hybridized with 10–50 short fluorescent DNA oligos tiling the target RNA; Multiple bound probes make each RNA appear as a bright, diffraction-limited spot that can be automatically detected and counted [21,22]. Nascent RNA at transcription sites appears as brighter nuclear foci, whose intensity (or inferred copy number) reports instantaneous transcriptional output, allowing estimates of burst size and frequency from single-cell distributions [23,24]. smFISH requires only a standard wide-field or confocal fluorescence microscope equipped with high-NA oil objectives (e.g., 40×–60×, NA 1.3–1.4) and a sensitive camera; Optional super-resolution or structured-illumination systems can further improve localization and 3D resolution but are not strictly necessary [21,24]. Applied to stem or progenitor cells, these methods have been used to reveal infrequent stochastic transcription bursts and cell-to-cell variability in key regulators, directly visualizing molecular noise at single-molecule resolution [23,24].

#### 2.2.2. Live-Cell RNA Imaging

Live-cell transcription imaging systems, such as MS2 and PP7 RNA-tagging, quantify transcriptional bursting by converting fluorescence at a single gene locus into a time-resolved record of nascent RNA synthesis. Stem-loop sequences are inserted into a reporter or endogenous gene; fluorescent coat proteins bind these loops as RNA polymerase II transcribes, producing a bright spot whose intensity is proportional to the number of actively transcribed RNA molecules [25,26].

Time-lapse microscopy tracks this spot in individual cells, generating fluorescence intensity traces over minutes to hours [25,26]. After calibration (often using smFISH or single-GFP standards), intensity is converted to numbers of nascent transcripts or engaged polymerases [26,27]. Bursts appear as episodes of elevated signal separated by low or no signal. By thresholding and quantitative analysis of these traces, one extracts burst frequency (events per unit time), burst duration (ON-period length), and burst amplitude/size (maximal or integrated nascent RNA per burst) [25,26,27,28]. Statistical or deconvolution methods then fit stochastic models (e.g., two-state promoters) to infer underlying kinetic parameters of bursting in living cells [27,28].

#### 2.2.3. scRNA-Seq Inference Approaches

Single-cell RNA sequencing (scRNA-seq) enables genome-wide inference of transcriptional bursting parameters across thousands of genes simultaneously, even in the absence of live imaging. scRNA-seq inference approaches quantify transcriptional bursting by fitting stochastic gene expression models (typically the two-state telegraph model) to single-cell count distributions across thousands of cells. Mature mRNA counts (often UMI-based) are modeled using mixture distributions (e.g., Beta-Poisson or Beta-Gamma-Poisson), from which the mean and variance are mapped to burst frequency and burst size for each gene [29].

Modern methods explicitly correct for technical noise, including dropouts, capture efficiency, and cell size variation, to avoid overestimating intrinsic noise [30,31]. Bayesian or likelihood-free frameworks (e.g., Approximate Bayesian Computation, neural-network-based simulators, or BISC) use simulations to match observed scRNA-seq histograms and provide genome-wide kinetic parameter estimates with confidence intervals [30,32].

#### 2.2.4. Spatial Transcriptomics

Spatial transcriptomics enables the indirect quantification of transcriptional bursting by measuring spatially resolved mRNA counts and fitting bursting models to the distribution of counts across cells or spatial spots. Imaging-based methods (e.g., MERFISH, seqFISH) detect individual transcripts as diffraction-limited spots, providing single-molecule, subcellular maps of RNA abundance that reveal heterogeneous, non-Poisson expression consistent with bursts [33,34]. Sequencing-based platforms capture counts per spatial spot, which can be deconvolved and analyzed with statistical and machine-learning tools to identify spatial domains of high or low burst activity and mean–variance relationships indicative of burst size and frequency [35,36]. Although still in the early stages, spatial transcriptomics holds promise for revealing how the stem cell niche modulates bursting parameters in pluripotent stem cells.

#### 2.2.5. Mathematical Modeling and Noise Quantification

The experimental approaches described above—ranging from smFISH and live-cell imaging to scRNAseq and spatial transcriptomics—yield raw measurements such as mRNA counts or fluorescence intensity traces. Mathematical modeling is required to convert these data into interpretable bursting parameters. In the two-state (telegraph) model, the promoter switches between inactive (OFF) and active (ON) states, with transcription only occurring in the ON state [7,13]. At steady state, this model yields a Beta-Poisson distribution (often approximated by a negative binomial in the bursty limit), allowing estimation of burst size and burst frequency from smFISH or scRNA-seq data using likelihood-based or Bayesian inference [7,30,31,37].

Beyond the canonical two-state model, three-state and kinetic proofreading frameworks provide deeper mechanistic insight into transcriptional bursting. Three-state models introduce an intermediate “poised” or “reloading” promoter state, capturing multi-step activation (e.g., chromatin remodeling or sequential transcription factor binding) [6,20,38]. This extension explains non-exponential ON/OFF dwell times observed in live-cell imaging and helps to distinguish transient silence from long-term repression, offering a more realistic view of developmental gene regulation [6,20,38].

The kinetic proofreading model addresses the specificity-speed paradox: transcription factors (TFs) must rapidly locate correct targets amid excess nonspecific DNA. By incorporating ATP-dependent, irreversible delay steps, such as chromatin remodeling or TFIIH activity, the system amplifies small differences in binding duration [6,39]. Only factors that persist through this energy-consuming checkpoint trigger transcriptional activation [1,6]. This non-equilibrium mechanism inherently generates bursty dynamics and decodes transcription factor dwell time into burst frequency [1,6,39]. Together, these models bridge molecular mechanisms, such as ATP hydrolysis and intermediate states, to observe bursting kinetics, enabling quantitative predictions for pluripotent stem cell regulation.

Noise quantification and dynamical model calibration/correction proceed by matching measured distributions and moments (Fano factor, CV^2^, higher moments) while explicitly modeling extrinsic variability (cell size, capture efficiency, cell-to-cell parameter variation) and post-transcriptional noise [30,31,40]. Recent work derives analytic bias signatures caused by extrinsic noise and uses them to correct inferred burst size and frequency, while master-equation solvers, reference-library pipelines, and simulation-based methods assess parameter identifiability and test whether two-state, multi-state, or generalized schemes are consistent with the data [31,40].

#### 2.2.6. Comparative Summary of Bursting Quantification Methods

We summarize the distinct advantages, limitations, and optimal use contexts of the methods for quantifying transcriptional bursting (Table 1). In brief, smFISH provides fixed-cell, single-molecule resolution with standard equipment, ideal for validating bursting parameters in defined cell populations, but lacks temporal information [23,32,41]. Live-cell imaging (MS2/PP7) offers real-time tracking of individual alleles, enabling direct measurement of burst duration and frequency, yet requires genetic engineering and is largely limited to reporter loci [25,39,42]. scRNA-seq enables genome-wide burst parameter estimation without imaging but relies on model assumptions and indirect inference [30,32,37,43,44]. Spatial transcriptomics adds tissue context but currently lacks single-allele resolution [34,45,46]. Researchers should select methods based on their specific question: live-cell imaging is good for mechanistic dissection of burst [25,39,42]; scRNA-seq is suitable for population-scale or differentiation studies [30,32,37,43,44]; smFISH is favored for validation of candidate genes [41,42,43].

## 3. Transcriptional Bursting in Pluripotent Stem Cells: A Special Case

### 3.1. Why PSCs Are an Ideal Model to Study Bursting

PSCs offer unparalleled advantages for studying transcriptional bursting. First, their open chromatin and high transcriptional activity produce widespread bursting across the genome, enabling robust genome-wide inference of bursting kinetics [10,13]. Second, their homogeneous state minimizes extrinsic noise from cell cycle or metabolism, ensuring that scRNA-seq or smFISH observations reliably reflect intrinsic promoter dynamics, thereby improving parameter estimation [13,15,47]. Third, well-defined state transitions during differentiation and reprogramming allow direct linkage of burst frequency and size to chromatin accessibility and epigenetic modifications [12,13,48].

Beyond these foundational strengths, PSCs are particularly powerful for mechanistic validation. Their high efficiency in homology-directed repair and robust transfection efficiency make them exceptionally amenable to genome editing, so CRISPR-based perturbations can be used for direct causal testing of bursting regulators [49,50,51]. Their uniform morphology and rapid proliferation also render PSCs well suited for live-cell imaging platforms (e.g., MS2-MCP), enabling real-time visualization of burst dynamics with high temporal resolution [11,26,52]. In addition, PSCs provide a tractable system for investigating allele-specific bursting: female PSCs display a defined X-chromosome inactivation status, and homologous chromosomes often exhibit clear epigenetic asymmetries (e.g., histone modifications and DNA methylation), creating a natural context to examine how parental-specific regulatory landscapes influence burst frequency and size [53,54]. Finally, patient-derived iPSCs link basic bursting mechanisms to human pathology [55,56]. Together, these features position PSCs as a premier model for dissecting how transcriptional bursts encode cell-fate decisions.

### 3.2. Unique Bursting Signatures of Core Pluripotency Genes

Oct4, Sox2 and Nanog are master TFs that establish and safeguard pluripotency in PSCs [57,58]. These core pluripotency genes exhibit distinctive transcriptional bursting dynamics. Single-cell and live-cell imaging studies have shown that they are transcribed in short, intermittent bursts rather than constitutively, thereby generating significant cell-to-cell expression heterogeneity [58,59]. For example, live imaging of Sox2 transcription revealed that bursting does not strictly require enhancer–promoter proximity, supporting a non-contact mode of enhancer function [59]. Their expression levels and bursting kinetics differ significantly: Oct4 displays relatively high and stable expression, whereas Nanog shows greater dynamic fluctuations and lower mean expression [60,61]. In addition, chromatin state, epigenetic modifications and 3D genome architecture strongly modulate burst frequency and allelic coordination [10,62]. Oct4, Sox2, and Nanog cooperate extensively in the pluripotency network [57,58]. In mESCs, Nanog shows pronounced reversible fluctuations and near-bimodal expression, with low-Nanog cells more prone to differentiation, suggesting that Nanog dynamics contributes to metastable states and population heterogeneity [15,58,60]. By contrast, Oct4 expression is relatively stable [60]. Live-imaging studies have directly shown that LIF-STAT3 signaling modulates Sox2 burst frequency and size, and that condensate proximity at the Sox2 super-enhancer enhances both parameters [11,63]. However, a direct, quantitative comparison of burst frequency and size among Oct4, Sox2, and Nanog has not been established. Thus, the unique bursting patterns of Oct4, Sox2 and Nanog not only maintain pluripotency but also underpin the molecular regulation of cell fate decisions.

### 3.3. Bursting Heterogeneity as a Driver of Cell-to-Cell Variability in PSCs

In PSCs, transcriptional bursting is a primary source of cell-to-cell expression variability [64]. Because bursting is stochastic, even genetically identical PSCs exhibit substantial differences in mRNA counts per cell [13,15]. For instance, Nanog exhibits infrequent, high-amplitude bursts, leading to a broad protein distribution that supports metastable pluripotent states [15]. Additionally, allele-specific bursting—where the two homologous alleles fire independently—further increases heterogeneity [10,15,65]. Importantly, this burst-driven variability is not just random noise; it allows PSCs to explore various expression landscapes, facilitating lineage priming and adaptive responses to differentiation signals while maintaining the pluripotent ground state [64,65]. Therefore, bursting heterogeneity directly influences the functional plasticity of PSCs.

## 4. Core Regulatory Mechanisms of Transcriptional Bursting in PSCs

**Figure 1:**
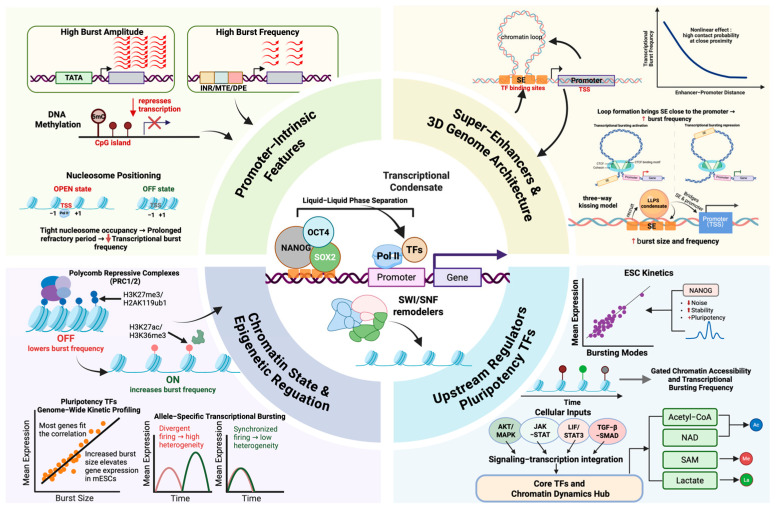
Core Regulatory Mechanisms of Transcriptional Bursting in PSCs.

Transcriptional bursting in PSCs is governed by a complex interplay of promoter-intrinsic features, enhancer–promoter communication, phase-separated condensates, chromatin state, and 3D genome architecture (Figure 1). Below, each regulatory layer is expanded with mechanistic detail and recent evidence.

### 4.1. Promoter-Intrinsic Features

The core promoter—the region where RNA polymerase II (Pol II) assembles the pre-initiation complex—carries intrinsic sequence features that directly influence bursting parameters. In PSCs, promoters of highly bursty genes (e.g., Nanog, Oct4) often contain a canonical TATA box or a CpG island with specific nucleosome-depleted regions [64]. The TATA box predominantly influences burst size, whereas Inr/MTE/DPE mainly modulate burst frequency and initiation timing. This was shown by systematic live-imaging analysis in Drosophila embryos, where TATA mutations reduce burst size, and Inr/MTE/DPE mutations primarily affect burst frequency and onset kinetics [38,66]. Moreover, promoter DNA methylation at CpG islands is known to repress transcription by reducing transcription factor accessibility, as extensively documented at the level of mean expression [67,68,69]. Although hypomethylated promoters correlate with higher transcriptional activity, direct evidence that DNA methylation suppresses burst frequency in PSCs remains lacking. Single-cell studies have further shown that promoter-proximal nucleosome positioning—particularly the −1 and +1 nucleosomes—creates a physical barrier that determines the duration of the OFF state [70]; tighter nucleosome occupancy prolongs OFF intervals, lowering burst frequency [1,13,70]. Thus, promoter-intrinsic features set the baseline bursting propensity upon which enhancer and chromatin regulators act.

### 4.2. Super-Enhancers

Enhancers—especially super-enhancers (SEs)—are critical for modulating transcriptional burst frequency. SEs are clusters of enhancers densely occupied by pluripotency TFs such as OCT4, SOX2, and NANOG at key loci [71,72]. Live-cell imaging and single-molecule studies reveal that enhancer–promoter proximity increases both burst frequency and size, though the effect is more pronounced on frequency [13,43]. The physical distance between enhancer and promoter inversely correlates with burst frequency; closer proximity or increased contact probability leads to more frequent bursts [63,73,74]. However, this relationship can be nonlinear due to chromatin topology and insulation by architectural proteins such as CTCF [74]. The Sox2/Oct4/Nanog loci exemplify these principles: their expression is tightly regulated by SEs through dynamic looping interactions that facilitate high-frequency bursting essential for maintaining pluripotency [63,71,75]. Notably, some studies suggest that direct spatial proximity is not always required for enhancer function; transient or stochastic contacts may suffice to trigger bursts [59].

### 4.3. Phase-Separated Transcriptional Condensates

The discovery of liquid–liquid phase separation (LLPS) has fundamentally changed our understanding of transcription regulation [76,77]. Transcriptional condensates are dynamic, membraneless compartments that concentrate TFs, co-activators, and Pol II at SEs and promoters [63,78]. Many pluripotency TFs (Oct4, Sox2, Nanog, Klf4) contain intrinsically disordered regions (IDRs) that mediate multivalent interactions and can induce phase separation [76,79]. These IDRs contribute to condensate formation at SEs in PSCs, and perturbing OCT4 phase separation impairs higher-order chromatin reorganization, although the precise impact on transcriptional bursting parameters is still being studied [80]. Condensate formation is driven by networks of multivalent interactions involving IDRs and is dynamically regulated by post-translational modifications and signaling-dependent changes in the molecular environment, enabling rapid responses to cellular signals [81,82,83].

Recent evidence has linked endogenous condensates to bursting parameters. Du et al. developed live-cell super-resolution and multi-color 3D-imaging approaches to investigate the role of endogenous condensates at the Sox2 SE in mouse embryonic stem cells [63]. They found that positional dynamics of the condensate, rather than the distance from the enhancer, better predict gene expression. When the condensate is more than 1 μm away, Sox2 exhibits basal bursting, but when it moves closer than 1 μm, both burst size and burst frequency increase. Disruptions to cohesin or local DNA elements do not eliminate basal bursting but compromise the condensate’s burst-enhancing ability [76]. Based on these observations, the authors proposed a “three-way kissing model” where the condensate transiently interacts with both the gene locus and regulatory DNA elements to control bursting. These findings establish a direct mechanistic connection between condensate dynamics and the modulation of burst size and frequency.

### 4.4. Chromatin State and Epigenetic Regulation

Chromatin accessibility is a prerequisite for transcriptional bursting. The positions of nucleosomes flanking the TSS influence OFF-state duration [84,85]. In yeast, remodeling of TSS-flanking nucleosomes by SWI/SNF-family remodelers is associated with increased transcription initiation frequency (a proxy for burst frequency) [85,86], but direct evidence in PSCs is lacking. In PSCs, esBAF is enriched at promoters of pluripotency genes and required for their expression, but its quantitative impact on transcriptional bursting parameters remains to be quantified [87]. Histone modifications—such as H3K27ac at enhancers/promoters or H3K36me3 within gene bodies—correlate with increased chromatin accessibility and higher burst frequencies [10,88,89]. Conversely, Polycomb repressive complexes (PRC1/2) deposit repressive marks like H3K27me3/H2AK119ub1 that compact chromatin structure and specifically suppress burst frequency without necessarily altering accessibility [90].

Allele-specific chromatin states further fine-tune bursting: coordinated allelic accessibility leads to synchronized bursting between alleles; divergent states promote independent firing and greater cell-to-cell heterogeneity [10,89].

### 4.5. Other 3D Genome Architecture

In pluripotent stem cells (PSCs), the genome is organized into topologically associating domains (TADs) that act as insulated regulatory neighborhoods, enriching enhancer–promoter contacts within the same domain [91,92]. Cohesin-mediated loop extrusion, halted orientation-dependently at CTCF sites, generates these TADs; clustered CTCF motifs at boundaries confer robust but incomplete insulation [91,92,93,94]. Consequently, boundaries are permeable: they provide only ~2-fold enrichment of intra- versus inter-domain contacts, and CTCF perturbation can rewire enhancer–promoter interactions in certain contexts [92,93,94].

How this 3D architecture shapes transcriptional bursting in PSCs is not directly established. Quantitative enhancer-relocation and modeling studies in non-PSC systems show that enhancer–promoter contact probability influences transcription nonlinearly, consistent with transient contacts being integrated into bursting dynamics [74]. Genome-wide analyses suggest that enhancers primarily encode burst frequency, while core promoters tune burst size [43,73]. In mouse ESCs, scRNA-seq and perturbation studies further implicate promoter- and gene-body-bound factors and signaling pathways in setting burst parameters, indicating additional regulatory layers beyond topology [13]. However, limited studies have demonstrated the association of specific TAD features (e.g., intra-domain contact frequency, boundary permeability, or cohesin/CTCF dosage) with corresponding changes in burst [59,91,92].

Phase-separated condensates and 3D folding are conceptually linked, with recent work showing that CTCF can mediate long-range compartment interactions via phase separation in PSCs [95], and that phase-separating transcription factors drive large-scale 3D genome reorganization during totipotent-like transitions [96]. Nevertheless, direct evidence that condensates quantitatively modulate cohesin/CTCF-mediated architecture to reshape bursting kinetics at defined PSC loci is still lacking. Thus, while TADs and loop extrusion clearly bias enhancer–promoter communication, their precise quantitative impact on burst frequency and size—and any condensate-mediated coupling—remains an open question requiring targeted, locus-resolved experiments in PSCs.

## 5. Emerging Signaling and Metabolic Networks Modulating Bursting Behaviors

Transcriptional bursting kinetics are not determined solely by promoter-proximal sequences or chromatin contacts. Extracellular ligands and intracellular metabolites also have the potential to shape these pulses, although their precise mechanistic routes remain elusive. Here, we survey the current convergence of signaling cascades and metabolic flux on burst frequency and size in PSCs. Therefore, we explicitly separate established observations from speculative inferences, acknowledging that several pathways are discussed below.

### 5.1. Pluripotency Transcription Factors

As discussed in Section 3.2, Oct4, Sox2, and Nanog exhibit distinct bursting patterns. Here we focus on how these transcription factors modulate bursting kinetics at the protein level.

All three factors contain IDRs that can promote phase separation, albeit with different efficiencies. Sox2 has the strongest propensity to form condensates at its super-enhancer, where condensate proximity within 1 μm correlates with increased burst size and frequency [63]. Oct4 contributes to phase separation via higher-order chromatin reorganization, although its direct impact on bursting parameters is less characterized [80]. Nanog, despite being IDR-rich, appears to stabilize bursting through sustained enhancer–promoter contacts rather than through condensate dynamics [75,97].

These factors also engage in distinct feedback circuits. Nanog activates its own expression through autoregulatory loops that amplify burst amplitude and sustain metastability [58]. Oct4 and Sox2 lack such strong autoregulation; instead, their burst parameters are tuned by chromatin accessibility and external signaling (e.g., LIF/STAT3 for Sox2) [11,98].

Thus, despite cooperative DNA binding, Oct4, Sox2, and Nanog modulate bursting through partially distinct mechanisms: Sox2 primarily through condensate-controlled frequency and burst size tuning, Oct4 through chromatin-coupled initiation, and Nanog through autoregulatory feedback and interaction stabilization.

### 5.2. Signaling Pathways

Beyond core pluripotency TFs, extrinsic signaling pathways indirectly modulate transcriptional bursting in PSCs. They achieve this by reshaping the intrinsic transcriptional landscape of these cells. A genome-wide study conducted on mouse embryonic stem cells (mESCs) has yielded key insights. It revealed that transcriptional bursting kinetics are regulated by proteins that bind to promoters and gene bodies. Notably, the AKT/MAPK signaling pathway modulates transcriptional bursting through its regulation of transcription elongation efficiency [13]. Elongation control shapes transcriptional bursting via multiple mechanisms. Promoter-proximal pausing and P-TEFb-mediated pause release regulate initiated Pol II molecules to enter productive elongation, thereby influencing burst output and size [99,100,101]. DNA supercoiling generated during elongation can feed back on transcription, modulating both elongation and initiation and thereby giving rise to bursting, a mechanism first demonstrated in bacteria and also shown to constrain transcriptional bursting of neighboring genes in eukaryotes [100,102,103,104]. In PSCs, this supercoiling feedback between neighboring genes has also been directly demonstrated [105]. Theoretical and computational models further suggest that slower elongation and longer gene bodies, by prolonging the time Pol II occupies a locus, can lengthen effective ON-state duration and thereby lower the apparent burst frequency [106,107,108]. To date, no direct link has been established between these signaling pathways, transcriptional bursting, and the maintenance of PSC pluripotency. Most existing studies focus on pathway activation and regulation of pluripotency, providing limited evidence connecting pathway activity to specific burst parameters, and few studies have explored the transcriptional bursting axis in the context of pluripotency maintenance.

Researchers have proposed that JAK-STAT signaling regulates transcriptional bursting in cancer cells. This regulatory role is closely associated with cellular adaptive responses, such as drug resistance [109]. However, its specific role in PSCs and the maintenance of pluripotency remains unclear. Similarly, the LIF/STAT3 pathway is well-established as a key regulator to maintain PSC pluripotency [110]. Despite this clear functional role, no direct evidence has been identified to link this effect to transcriptional bursting. Additionally, TGFβ-SMAD signaling serves as a critical pathway in regulating PSC fate transitions. This pathway mainly exerts its regulatory effects by coordinating with DNA methylation to modulate the expression of pluripotency factors. However, no direct evidence connects TGFβ-SMAD signaling to transcriptional bursting in PSCs.

Given the lack of direct evidence, these signaling pathways can only be rigorously classified as indirect modulators of transcriptional bursting in PSCs. They mediate bursting through core TFs and chromatin dynamics, rather than directly governing the pulse process. Transcriptional condensates, for instance, are key mediators of transcriptional regulation. These condensates can be regulated by signaling pathways, which in turn further affect transcriptional bursting [81]. The regulatory role of JAK-STAT signaling in PSC bursting and pluripotency maintenance requires further confirmation. Similarly, whether LIF/STAT3 maintains PSC pluripotency through the modulation of bursting remains an unresolved question.

### 5.3. Metabolic Inputs

Metabolic inputs also hold the potential to regulate transcriptional bursting in PSCs. Among these metabolic inputs, acetyl-coenzyme A (Acetyl-CoA) stands out as a key candidate. Its candidacy stems from its critical role in linking cellular metabolism to epigenetic regulation. Acetyl-CoA functions as a key intermediate in cellular metabolism. It participates in energy metabolism and serves as a substrate for histone acetyltransferases [111]. Despite this well-established role, direct evidence connecting Acetyl-CoA or other metabolic inputs to the transcriptional bursting of pluripotency-related genes in PSCs is still lacking.

Beyond Acetyl-CoA, other metabolic inputs also possess the potential to regulate transcriptional bursting in PSCs, which may form a complex metabolic-transcriptional regulatory network. This network likely coordinates the maintenance of pluripotency and the determination of cell fate. For instance, nicotinamide adenine dinucleotide (NAD+) and S-adenosylmethionine (SAM) serve as notable examples. These two key metabolic intermediates are involved in various epigenetic modifications, including histone deacetylation and DNA methylation, that are closely associated with chromatin accessibility and transcriptional activity [112,113]. In addition, lactate-derived histone lactylation serves as another metabolic-epigenetic mechanism that directly modulates chromatin state and gene transcription, suggesting that diverse cellular metabolites may collectively shape transcriptional dynamics through chromatin modifications [114].

To date, most existing studies have focused on the regulatory role of metabolism in PSC pluripotency. Few studies, however, have directly investigated how metabolic inputs tune the kinetic characteristics of transcriptional bursting. This research gap underscores the necessity for further studies. These studies should dissect the specific molecular mechanisms underlying the crosstalk between metabolism, transcriptional bursting, and PSC pluripotency.

### 5.4. Extrinsic Conditions

Emerging evidence indicates that extrinsic conditions also modulate transcriptional bursting dynamics in pluripotent stem cells. For example, the Akt/MAPK signaling pathway regulates transcriptional elongation and reshapes bursting parameters in mESCs [13], and this pathway is modulated by exogenous factors in the culture medium, including FGF, serum, growth factors, and small-molecule inhibitors (such as MEK inhibitors under 2i culture conditions) [115,116,117]. Comparative transcriptome and binding landscape analyses of core pluripotency factors (OCT4/SOX2/NANOG) in mESCs cultured under serum/LIF versus 2i conditions reveal that 2i rapidly remodels OSN binding and enhancer activity, attenuates spontaneous differentiation, and establishes a more stable ground state [118], which may reshape transcriptional bursting. Furthermore, under 2i conditions, mESCs display more homogeneous expression of pluripotency-associated genes and a reorganized chromatin landscape [118,119,120]. These changes in gene expression heterogeneity and chromatin organization are consistent with, and in some contexts linked to, altered transcriptional bursting kinetics that may stabilize the expression of key regulators such as Nanog, although a direct increase in Nanog burst frequency under 2i has not yet been firmly established [13,119].

## 6. Functional Outcomes of Transcriptional Bursting in PSCs

The functional outcomes of transcriptional bursting may be linked to cell fate decisions in PSCs. First, bursting parameters may instruct cell fate by establishing quantitative thresholds and temporal patterns of key regulators (for example, graded Sox2 output maintains pluripotency, whereas a sustained reduction in Sox2 transcription is sufficient to trigger exit from the pluripotent state) [11,121,122]. Second, bursting may serve as an emergent readout of underlying chromatin and signaling states—such as PRC2 occupancy, elongation efficiency, or coordinated allelic accessibility—that track cell state without necessarily driving it [10,13,89]. Third, reciprocal feedback is plausible: pluripotent and reprogramming states both regulate burst kinetics (including allelic coordination), while altered bursting in turn fine-tunes gene dosage and state transitions during iPSC reprogramming [10,89].

In mESCs, LIF–STAT3 perturbation reduces Sox2 burst frequency and size, shortens Sox2-ON periods, and this partial reduction in Sox2 transcription is sufficient to induce early differentiation, revealing quantitative thresholds for pluripotency maintenance [11]. Signaling pathways such as Akt/MAPK modulate bursting via Pol II pause release, offering additional routes by which extrinsic cues can reshape fate-relevant intrinsic noise [13]. Furthermore, global studies of PSC heterogeneity and allelic bursting argue that many correlations between bursting and fate remain mechanistic associations rather than proven causes [10,14,89,123].

Future work should be carried out by combining live-cell measurements of bursting with acute, locus- and pathway-specific perturbations of burst frequency or size, followed by single-cell lineage tracing in PSCs and during reprogramming [10,11,13]. Such integrative approaches will clarify when burst kinetics truly drive, versus merely accompany, stem cell fate decisions.

### 6.1. Maintenance of Pluripotency and Noise Control

The functional link between transcriptional bursting and pluripotency maintenance represents a core focus in PSC research. Transcriptional bursting directly modulates the expression dynamics of core pluripotency genes, and this modulation is critical for sustaining the undifferentiated state of PSCs. Core pluripotency factors, including Oct4, Sox2, and Nanog, are essential for maintaining PSC pluripotency. These factors are characteristically expressed in a transcriptional bursting mode [111]. This bursting expression pattern is thought to be subject to regulatory control, a mechanism that ensures appropriate expression levels of these key factors. Insufficient expression of these core pluripotency factors has been linked to the transition from naive to primed pluripotency. In contrast, excessive expression may perturb cellular homeostasis, highlighting the importance of maintaining proper expression dosage for pluripotency maintenance [124].

Specifically, the burst frequency and burst size of these core pluripotency genes undergo dynamic modulation, which likely contribute to maintaining the appropriate expression levels required for sustaining the pluripotent state [125]. Despite the clear association between transcriptional bursting and pluripotency maintenance, direct evidence elucidating how specific burst parameters functionally regulate the pluripotent state is still emerging. Furthermore, the underlying molecular mechanisms governing this regulation require further in-depth investigation [126].

Notably, the specific molecular mechanisms through which transcriptional bursting regulates gene expression noise in PSCs remain largely unclear. Existing studies have confirmed an association between transcriptional bursting parameters and gene expression noise [125]. Even so, direct evidence is still lacking that would elucidate how PSCs precisely tune burst parameters to achieve noise control, and how this noise control process in turn supports pluripotency maintenance. This research gap indicates the need for additional studies. These studies should dissect the functional link between transcriptional bursting, noise control, and the undifferentiated state of PSCs [13].

### 6.2. Cell Fate Decision and Differentiation

Cell fate decision represents a fundamental functional process in PSCs. Within this process, transcriptional bursting is recognized as a key regulatory hub that dynamically shapes the expression profiles of both pluripotency-associated genes and lineage-specific genes [13,63,125]. The intrinsic stochasticity inherent to gene bursting confers PSCs with the flexibility to initiate cell fate transitions [13,15,64]. However, the precise control of bursting kinetics is hypothesized to ensure orderly progression and maintain cellular homeostasis [10].

When mESCs undergo naive-to-primed transition or early mesendoderm commitment, live-cell imaging can directly validate that the burst frequency of Sox2 and Nanog declines, which weakens pluripotency and facilitates fate transition [11,60]. By contrast, lineage-specific genes such as Brachyury are proposed to exhibit progressive increases in burst size during early differentiation, potentially promoting mesodermal commitment [43]. These coordinated changes are computationally predicted to be constrained by promoter-dependent feedback loops, yet not directly validated in PSCs [44]. Hence, transcriptional bursting is thought to regulate differentiation by modulating the dynamics of pluripotency and lineage genes [43].

In differentiating mESCs, Oct4 burst frequency is confirmed to decrease at late stages, further suppressing the undifferentiated state [11,21]. Lineage-specific genes exhibit distinct bursting tendencies during specification: ectodermal genes tend to increase burst size, whereas mesodermal genes are more likely to show elevated burst frequency [43,127,128]. In cardiac mesoderm differentiation of mESCs, cardiac pioneer factors such as Gata4 are temporally activated, which may be regulated by stage-specific burst frequency dynamics [129]. For neural differentiation of mESCs, neural precursor genes such as Nestin are upregulated, which may correlate with increased burst size to support neural lineage specification [128].

Genome-wide studies indicate that distinct transcriptional bursting landscapes are associated with different differentiation outcomes in mammalian PSCs [13,44,73,130]. Although a correlation between transcriptional bursting and PSC fate decision is widely proposed, direct experimental evidence remains limited [31,130].This gap largely stems from technical challenges in tracking burst parameters at single-cell resolution across continuous fate transitions [31].The molecular mechanisms linking burst kinetics to fate commitment, as well as how external signals (e.g., LIF, TGFβ) fine-tune bursting to guide fate choices, remain incompletely understood [110,131]. While single-cell transcriptomics has advanced our understanding of PSC fate dynamics, the regulatory role of bursting behavior has been poorly characterized [132].

### 6.3. Heterogeneity and Priming

Heterogeneity represents an inherent feature of PSC populations, even under undifferentiated culture conditions. It manifests as heterogeneous expression of pluripotency genes and lineage-specific genes across individual cells [133]. Far from random, this heterogeneity associates closely with the priming state of PSCs [129]. Priming describes the phenomenon whereby undifferentiated PSCs become predisposed to differentiate into specific cell lineages. This predisposition lays a foundation for the rapid initiation of lineage commitment once these cells receive external differentiation signals [132,134].

Transcriptional bursting is hypothesized to drive PSC heterogeneity and priming [125]. Variations in burst parameters across individual cells give rise to heterogeneous expression of pluripotency and lineage-associated genes. These expression differences, in turn, generate priming subpopulations within the PSC pool [135].

For example, within undifferentiated human PSC populations, a subset of cells displays elevated burst frequencies of mesoderm-specific genes, a signature indicative of mesodermal priming. In contrast, other cells exhibit enhanced burst parameters associated with ectoderm-specific loci, reflecting an ectodermal predisposition [136]. These modifications govern the accessibility of promoters and enhancers, which in turn modulate the burst kinetics of lineage-specific genes [137]. Enhancer–promoter (E-P) communication further modulates the bursting of priming-associated genes. The strength of E-P interactions follows a positive power law to up-regulate both burst frequency and size. This relationship amplifies lineage-specific priming. Conversely, the genomic distance separating E-P elements follows a negative power law to suppress bursting. This mechanism restricts the over-activation of lineage-specific genes, thereby preserving pluripotent potential while maintaining a primed state [73].

Current evidence directly connecting transcriptional bursting to PSC heterogeneity and priming remains conspicuously limited. This scarcity arises from technical challenges in tracking the dynamics at single-cell resolution across extended time periods, which is essential for establishing definitive causal inference. Furthermore, the precise molecular mechanisms by which external signaling pathways regulate burst dynamics to adjust the primed state and sculpt population heterogeneity remain largely uncharacterized [11].

## 7. Challenges and Future Perspectives

### 7.1. Challenges

Several limitations remain in this field. First, integrated live-cell imaging of endogenous bursting, condensates, and 3D genome architecture is currently restricted to only a few loci or fixed cells; a routine, genome-wide framework for causal analysis is lacking. Second, though allele-resolved burst kinetics have been quantified transcriptome-wide using allele-sensitive scRNA-seq, revealing distinct cis-regulatory control of burst size and frequency, their links to cellular heterogeneity and transcription factor binding remain incompletely mapped. Third, while perturbations can preferentially modulate burst frequency (e.g., via enhancers or histone acetylation) or burst size (e.g., via core promoters or MYC), these parameters are mechanistically coupled, and current tools do not enable precise, locus-specific, independent tuning. Finally, a validated quantitative roadmap connecting burst parameters to PSC quality, lineage commitment, or regenerative outcomes is still lacking.

### 7.2. Future Perspectives

As discussed above, for future studies in this field, we propose a staged, forward-looking roadmap.

Short-term: Develop CRISPR–dCas9 or optogenetic tools to selectively bias burst frequency or size at endogenous loci, rather than fully decoupling them. Establish exploratory reference atlases of transcriptional bursting for human pluripotent stem cell (PSC) lines, and test whether burst signatures (e.g., NANOG burst frequency or allelic synchrony) correlate with functional potency as candidate surrogates for PSC quality control.

Mid-term: Train machine learning models that explicitly incorporate burst parameters inferred from single-cell datasets of lineage-specifying transcription factors (e.g., BRACHYURY (T), PAX6, SOX17) to probabilistically predict single-cell differentiation trajectories. Use real-time bursting readouts to guide optimization of differentiation protocols, and screen for small molecules that modulate aberrant bursting patterns in patient-derived induced PSCs (iPSCs).

Long-term: Aspirational goals include in vivo monitoring of bursting dynamics in grafted cells during tissue regeneration and designing synthetic bursting circuits that bias “self-guided” differentiation decisions in vivo or in engineered niches. These forward-looking advances would ultimately enable targeted control of transcriptional noise, informing PSC quality management and lineage prediction in regenerative medicine.

## 8. Conclusions

Transcriptional bursting is a fundamental regulatory mechanism that controls gene expression heterogeneity, pluripotency maintenance, and cell fate commitment in pluripotent stem cells. This dynamic process is governed by a complex regulatory network. Core TFs, super-enhancers, and transcriptional condensates work together with chromatin states and metabolic inputs to tune burst frequency and size. Current research suggests that intrinsic noise drives individual gene expression fluctuations, whereas extrinsic factors contribute to variability across the cell population. Although technical barriers still hinder the direct manipulation of bursting, a unified framework can be established to link molecular kinetics with stem cell behavior. The integration of single-molecule imaging and genome engineering is expected to facilitate the precise control of transcriptional bursting for applications in regenerative medicine.

## Figures and Tables

**Table 1:** Comparison of methods for quantifying transcriptional bursting in PSCs.

Method	Temporal Resolution	Spatial Resolution	Throughout	Genetic Modification Needed	Key Strength	Main Limitation
smFISH	Snapshot (fixed cells)	Single mRNA/single cell	Low-medium (per gene set)	No	Direct single-molecule counting; no imaging reporters	No temporal dynamics; limited gene number per experiment
Live-cell imaging (MS2/PP7)	Seconds-minutes	Single allele (locus-specific)	Low	Yes (knock-in/tagging)	Real-time burst kinetics at defined loci	Locus-specific; requires genetic engineering and imaging infrastructure
scRNA-seq inference	Snapshot	Single cell (not allele-resolved)	High (genome-wide)	No	Genome-scale estimation of burst parameters without imaging	Indirect, model-dependent inference from static data
Spatial transcriptomics	Snapshot	Near-single-cell spatial resolution	Medium-high	No	Adds tissue/microenvironment context to transcriptional states	Limited allele resolution; methods still emerging for bursting analysis

## Data Availability

No new data were created or analyzed in this study.

## Abbreviations

The following abbreviations are used in this manuscript:

Acetyl-CoA	Acetyl-coenzyme A
iPSCs	Induced pluripotent stem cells
IDRs	Intrinsically disordered regions
LLPS	liquid–liquid phase separation
mESCs	Mouse embryonic stem cells
NAD+	Nicotinamide adenine dinucleotide
PSCs	Pluripotent stem cells
SAM	S-adenosylmethionine
scRNA-seq	Single-cell RNA sequencing
smFISH	Single-molecule fluorescence in situ hybridization
SEs	Super-enhancers
TADs	Topologically associating domains
TFs	Transcription factors
